# Supplementary material for: Language measures correlate with other measures used to study emotion
Source: Commun Psychol. 2025 Feb 22;3:29. doi: 10.1038/s44271-025-00212-x (PMC11847001; doi:10.1038/s44271-025-00212-x)
Supplement: Supplementary file 2 — Supplemental Material [file 44271_2025_212_MOESM2_ESM.pdf]

Language measures correlate with other measures used to study emotion

Supplementary Information

Table of contents:

Descriptive statistics for study variables (Tables S1a-S2)..………………………………………………………..………pp 2-6

Correlations for language measures of valence (Tables S3a-S3c)………………………………………..………………pp 7-11

Bonferroni corrections and CIs for language measures of valence (Tables S4a-S4d)……………………...……...………pp 12-19

Correlations for language measures of discrete emotions (Tables S5a-S5i)……...…………………...……………..…….pp 20-28

Bonferroni corrections and CIs for language measures of discrete emotions (Tables S6a-S6d)……………………..……pp 29-35

Comparison of association patterns between dictionaries & Bonferroni corrections (Tables S7a-S8)...……………………..pp 36-38

Descriptive statistics for language measures of emotion frequency and intensity (Supplementary Note 1; Table S9)………pp 39-40

Correlations for language measures of emotion frequency and intensity (Tables S10a-S11c)………………………………pp 41-49

Table S1a.

Descriptive statistics for self-report measures in Dataset 1 (social rejector narratives)

	M	SD	Range
Pos-Neg Self-Report	-0.63	1.13	-3.15–3.59
Angry Self-Report	1.82	1.13	1–5
Annoyed Self-Report	2.77	1.40	1–5
Worried Self-Report	2.86	1.37	1–5
Self-Conscious Self-Report	2.46	1.28	1–5
Sad Self-Report	2.04	1.13	1–5
Hurt Self-Report	1.81	1.20	1–5

Table S1b.

Descriptive statistics for self-report, observer report, facial cue, and vocal measures in Dataset 2 (SEND narratives)

	M	SD	Range
Self-Report	55.45	20.97	3.13–95.44
Observer Report	51.79	17.51	16.51–84.13
Happy Face	-1.13	1.46	-5.05–2.72
Angry Face	-2.26	1.06	-4.69–0.05
Fearful Face	-1.67	0.78	-4.00–0.19
Sad Face	-1.59	0.97	-5.05–0.66
Vocal Pitch	24.48	3.07	17.57–30.76
Vocal Intensity	0.97	0.28	0.41–1.85

Table S1c.

Descriptive statistics for self-report, facial cue, and vocal measures in Dataset 3 (CANDOR conversations)

	M	SD	Range
Pre-Task Self-Report	6.10	1.50	1–9
Retrospective Self-Report	7.32	1.30	1–9
Post-Task Self-Report	7.30	1.32	1–9
Happy Face	0.33	0.18	0.00–0.90
Angry Face	0.07	0.07	0.00–0.60
Fearful Face	0.05	0.05	0.00–0.36
Sad Face	0.12	0.09	0.00–0.74
Vocal Pitch	189.85	39.30	107.32–287.03
Vocal Intensity	0.77	0.05	0.41–0.91

Table S2.

Descriptive statistics for language measures in Datasets 1 (rejector narratives), 2 (SEND narratives), and 3 (CANDOR conversations)

	**Dataset 1**			**Dataset 2**			**Dataset 3**		
	M	SD	Range	M	SD	Range	M	SD	Range
**Valence**									
LIWC Tone	33.08	23.43	1.00–97.37	41.69	27.85	1.00–99.00	48.82	13.28	8.67–93.61
NRC Pos-Neg	1.54	1.97	-6.00–10.06	1.91	2.04	-2.33–8.47	1.99	0.89	-0.99–5.91
LS Valence	4.48	1.57	0.85–8.45	5.33	1.69	1.27–8.45	5.28	0.77	2.56–7.66
ANEW Valence	6.13	0.43	3.47–7.63	6.46	0.21	5.28–7.34	6.55	0.18	5.71–7.10
VADER Compound	0.41	0.74	-1.00–1.00	0.74	0.60	-1.00–1.00	1.00	0.03	-0.83–1.00
VADER Pos-Neg	0.03	0.06	-0.20–0.22	0.10	0.09	-0.10–0.33	0.23	0.07	0.01–0.54
**Discrete Emotions**									
LIWC Anger	0.17	0.36	0.00–2.80	0.08	0.22	0.00–1.59	0.06	0.07	0.00–0.87
NRC Anger	0.95	0.83	0.00–8.40	0.80	0.69	0.00–3.80	0.68	0.30	0.00–2.67
NRC Anger Intensity	0.46	0.13	0.06–0.85	0.42	0.17	0.01–0.75	0.44	0.08	0.06–0.78
LIWC Anxiety	0.13	0.30	0.00–2.50	0.18	0.34	0.00–2.41	0.09	0.10	0.00–1.20
NRC Fear	1.00	0.85	0.00–6.52	1.00	0.79	0.00–3.90	0.87	0.35	0.06–2.59

NRC Fear Intensity	0.42	0.13	0.03–0.73	0.42	0.15	0.03–0.84	0.43	0.07	0.14–0.69
LIWC Sadness	0.14	0.29	0.00–2.40	0.24	0.48	0.00–4.35	0.04	0.05	0.00–0.51
NRC Sadness	1.26	0.97	0.00–9.16	1.14	0.88	0.00–4.15	0.75	0.29	0.00–2.15
NRC Sadness Intensity	0.45	0.14	0.00–0.84	0.45	0.16	0.02–0.89	0.42	0.07	0.04–0.71

Table S3a.

Spearman correlations between language measures of valence and other measures in Dataset 1 (social rejector narratives)

	1.	2.	3.	4.	5.	6.	7.
1. Valence Self-Report	—	.18*	.07	.12*	.20*	.12*	.18*
2. LIWC Tone		—	.45*	.58*	.51*	.48*	.53*
3. NRC Pos-Neg			—	.33*	.42*	.43*	.45*
4. LS Valence				—	.41*	.52*	.56*
5. ANEW Valence					—	.39*	.44*
6. VADER Compound						—	.90*
7. VADER Pos-Neg							—

Note. *N*s = 588-602. Asterisks indicate $p < .05$.

Table S3b.

Spearman correlations between language measures of valence and other measures in Dataset 2 (SEND narratives)

	1.	2.	3.	4.	5.	6.	7.	8.	9.	10.	11.	12.	13.	14.
1. Valence Self-Report	—	.80*	.17*	-.19*	-.09	-.14*	.07	.08	.64*	.52*	.62*	.51*	.51*	.60*
2. Valence Observer Report		—	.25*	-.15*	-.09	-.24*	.13	.10	.71*	.60*	.73*	.55*	.59*	.68*
3. Happy Face			—	-.23*	.00	-.50*	.25*	.00	.09	.10	.15*	.24*	.26*	.27*
4. Angry Face				—	.15*	.38*	-.39*	.03	-.08	.05	-.13	-.12	-.20*	-.17*
5. Fearful Face					—	.27*	-.16*	-.10	-.08	-.05	-.02	-.07	-.10	-.10
6. Sad Face						—	-.36*	.00	-.03	-.07	-.10	-.08	-.20*	-.11
7. Vocal Pitch							—	.43*	.06	.02	.13	.07	.25*	.17*
8. Vocal Intensity								—	.00	-.06	.10	.08	.11	-.01
9. LIWC Tone									—	.69*	.71*	.56*	.55*	.72*
10. NRC Pos-Neg										—	.57*	.41*	.41*	.55*
11. LS Valence											—	.52*	.54*	.62*
12. ANEW Valence												—	.61*	.71*
13. VADER Compound													—	.87*

14. VADER Pos-Neg	—

Note. $N = 193$. Asterisks indicate $p < .05$.

Table S3c.

Spearman correlations between language measures of valence and other measures in Dataset 3 (CANDOR conversations)

	1.	2.	3.	4.	5.	6.	7.	8.	9.	10.	11.	12.	13.	14.
1. Pre-Task Valence	—	.35*	.40*	-.08*	.13*	.13*	.05	-.02	.01	.05*	.02	.07*	-.08*	-.10*
2. Retrospective Valence	.37*	—	.77*	-.05	.13*	.13*	.02	.03	.06*	.00	-.03	.02	-.04	-.13*
3. Post-Task Valence	.44*	.84*	—	-.06*	.14*	.13*	.03	.05	.09*	-.03	-.05	.01	-.05*	-.14*
4. Happy Face	-.06*	.02	.03	—	-.50*	-.44*	-.48*	.20*	.14*	.10*	.06*	.14*	.18*	.22*
5. Angry Face	.09*	.08*	.07*	-.51*	—	.22*	.02	-.17*	-.12*	-.06*	-.04	-.10*	-.18*	-.24*
6. Fearful Face	.08*	.11*	.09*	-.47*	.27*	—	.27*	.01	.04	-.02	-.03	-.06*	-.10*	-.19*
7. Sad Face	.04	.03	.01	-.46*	.07*	.27*	—	-.08*	-.07*	-.03	-.04	-.05	-.13*	-.12*
8. Vocal Pitch	-.06*	.05*	.07*	.24*	-.19*	-.04	-.09*	—	.79*	.08*	-.07*	.12*	.00	.04
9. Vocal Intensity	-.02	.10*	.12*	.15*	-.12*	.02	-.09*	.80*	—	.00	-.06*	.05*	.00	-.04
10. LIWC Tone	.02	.01	.01	.13*	-.06*	-.05*	-.02	.08*	-.02	—	.59*	.67*	.20*	.42*
11. NRC Pos-Neg	.01	-.02	-.02	.10*	-.08*	-.06*	-.07*	-.03	-.04	.58*	—	.47*	.33*	.31*
12. LS Valence	.05*	.04	.06*	.14*	-.10*	-.07*	-.02	.14*	.05*	.63*	.45*	—	.14*	.40*
13. ANEW Valence	-.03	-.01	.00	.23*	-.21*	-.15*	-.16*	.06*	.04	.26*	.33*	.19*	—	.58*

14. VADER Pos-Neg	-.07*	-.08*	-.05*	.26*	-.26*	-.24*	-.10*	.14*	.00	.42*	.30*	.40*	.62*	—

Note. First-time speakers (Dataset 3a) above the diagonal (*N*s = 1406-1455). Returning speakers (Dataset 3b) below the diagonal (*N*s = 1837-1856). Asterisks indicate $p < .05$.

Table S4a.

Bonferroni corrected p-values and 95% CIs for correlations between language measures of valence and self-report

Dataset	Self-report measure	Language measure	Spearman's rho	95% CI	Original p-value	Bonferroni-corrected p-value
1	Pos-neg self-report	LIWC Tone	.18	[.10, .26]	< .001	< .001
		NRC Pos-Neg	.07	[-.01, .15]	.079	.475
		LS Valence	.12	[.04, .20]	.004	.024
		ANEW Valence	.20	[.13, .28]	< .001	< .001
		VADER Compound	.12	[.04, .20]	.004	.024
		VADER Pos-Neg	.18	[.10, .26]	< .001	< .001
2	Self-report	LIWC Tone	.64	[.54, .71]	< .001	< .001
		NRC Pos-Neg	.52	[.41, .61]	< .001	< .001
		LS Valence	.62	[.53, .70]	< .001	< .001
		ANEW Valence	.51	[.40, .61]	< .001	< .001
		VADER Compound	.51	[.40, .61]	< .001	< .001
		VADER Pos-Neg	.60	[.50, .68]	< .001	< .001
3a	Pre-task self-report	LIWC Tone	.05	[.00, .10]	.042	.211
		NRC Pos-Neg	.02	[-.03, .07]	.448	> .99
		LS Valence	.07	[.02, .12]	.005	.026
		ANEW Valence	-.08	[-.13, -.03]	.002	.011
		VADER Pos-Neg	-.10	[-.15, -.04]	< .001	.002
	Retrospective self-report	LIWC Tone	.00	[-.05, .06]	.874	> .99
		NRC Pos-Neg	-.03	[-.08, .03]	.312	> .99
		LS Valence	.02	[-.03, .07]	.477	> .99
		ANEW Valence	-.04	[-.09, .01]	.110	.548
		VADER Pos-Neg	-.13	[-.18, -.08]	< .001	< .001
	Post-task self-report	LIWC Tone	-.03	[-.08, .03]	.341	> .99
		NRC Pos-Neg	-.05	[-.10, .01]	.082	.412
		LS Valence	.01	[-.05, .06]	.809	> .99
		ANEW Valence	-.05	[-.11, -.00]	.042	.212

		VADER Pos-Neg	-.14	[-.19, -.08]	< .001	< .001
3b	Pre-task self-report	LIWC Tone	.02	[-.02, .07]	.291	> .99
		NRC Pos-Neg	.01	[-.04, .05]	.757	> .99
		LS Valence	.05	[.00, .09]	.046	.230
		ANEW Valence	-.03	[-.08, .02]	.197	.986
		VADER Pos-Neg	-.07	[-.12, -.03]	.002	.012
	Retrospective self-report	LIWC Tone	.01	[-.04, .05]	.715	> .99
		NRC Pos-Neg	-.02	[-.07, .02]	.309	> .99
		LS Valence	.04	[-.01, .09]	.089	.444
		ANEW Valence	-.01	[-.06, .03]	.587	> .99
		VADER Pos-Neg	-.08	[-.12, -.03]	< .001	.005
	Post-task self-report	LIWC Tone	.01	[-.03, .06]	.545	> .99
		NRC Pos-Neg	-.02	[-.06, .03]	.493	> .99
		LS Valence	.06	[.01, .11]	.010	.0495
		ANEW Valence	.00	[-.05, .04]	.924	> .99
		VADER Pos-Neg	-.05	[-.10, -.01]	.023	.115

Note. Number of comparisons = 6 for Datasets 1 and 2. Number of comparisons = 5 for Dataset 3. CI = confidence interval. Alpha level = .05

Table S4b.

Bonferroni corrected p-values and 95% CIs for correlations between language measures of valence and observer report

Dataset	Observer report measure	Language measure	Spearman's rho	95% CI	Original p-value	Bonferroni-corrected p-value
2	Observer report	LIWC Tone	.71	[.63, .77]	< .001	< .001
		NRC Pos-Neg	.60	[.50, .69]	< .001	< .001
		LS Valence	.73	[.65, .79]	< .001	< .001
		ANEW Valence	.55	[.45, .64]	< .001	< .001
		VADER Compound	.59	[.49, .68]	< .001	< .001
		VADER Pos-Neg	.68	[.59, .75]	< .001	< .001

Note. Number of comparisons = 6. CI = confidence interval. Alpha level = .05

Table S4c.

Bonferroni corrected p-values and 95% CIs for correlations between language measures of valence and facial cues

Dataset	Facial cue measure	Language measure	Spearman's rho	95% CI	Original p-value	Bonferroni-corrected p-value
2	Happy face	LIWC Tone	.09	[-.05, .23]	.215	> .99
		NRC Pos-Neg	.10	[-.04, .29]	.172	> .99
		LS Valence	.15	[.01, .29]	.034	.206
		ANEW Valence	.24	[.10, .37]	.001	.005
		VADER Compound	.26	[.12, .38]	< .001	.002
		VADER Pos-Neg	.27	[.13, .39]	< .001	.001
	Negative face	LIWC Tone	-.09	[-.23, .05]	.191	> .99
		NRC Pos-Neg	.01	[-.13, .15]	.909	> .99
		LS Valence	-.12	[-.26, .02]	.093	.560
		ANEW Valence	-.14	[-.28, -.00]	.047	.282
		VADER Compound	-.28	[-.41, -.15]	< .001	< .001
		VADER Pos-Neg	-.20	[-.34, -.07]	.004	.026
	Angry face	LIWC Tone	-.08	[-.22, .07]	.289	> .99
		NRC Pos-Neg	.05	[-.09, .19]	.482	> .99
		LS Valence	-.13	[-.26, .02]	.083	.499
		ANEW Valence	-.12	[-.26, .02]	.093	.558
		VADER Compound	-.20	[-.33, -.06]	.006	.036
		VADER Pos-Neg	-.17	[-.30, -.03]	.021	.127
	Fearful face	LIWC Tone	-.08	[-.22, .06]	.257	> .99
		NRC Pos-Neg	-.05	[-.19, .09]	.479	> .99
		LS Valence	-.02	[-.16, .12]	.739	> .99
		ANEW Valence	-.07	[-.21, .07]	.324	> .99
		VADER Compound	-.10	[-.24, .04]	.162	.970
		VADER Pos-Neg	-.10	[-.24, .04]	.165	.988
	Sad face	LIWC Tone	-.03	[-.17, .11]	.677	> .99
		NRC Pos-Neg	-.07	[-.21, .07]	.329	> .99
		LS Valence	-.10	[-.24, .04]	.159	.955

		ANEW Valence	-.08	[-.22, .06]	.284	> .99
		VADER Compound	-.20	[-.33, -.06]	.005	.029
		VADER Pos-Neg	-.11	[-.25, .03]	.114	.684
3a	Happy face	LIWC Tone	.10	[.05, .15]	< .001	< .001
		NRC Pos-Neg	.06	[.01, .11]	.019	.094
		LS Valence	.14	[.09, .19]	< .001	< .001
		ANEW Valence	.18	[.13, .23]	< .001	< .001
		VADER Pos-Neg	.22	[.17, .27]	< .001	< .001
	Negative face	LIWC Tone	-.06	[-.11, -.01]	.017	.086
		NRC Pos-Neg	-.05	[-.10, -.00]	.047	.237
		LS Valence	-.10	[-.15, -.05]	< .001	.001
		ANEW Valence	-.21	[-.26, -.16]	< .001	< .001
		VADER Pos-Neg	-.25	[-.29, -.20]	< .001	< .001
	Angry face	LIWC Tone	-.06	[-.11, -.01]	.018	.088
		NRC Pos-Neg	-.04	[-.09, .01]	.105	.524
		LS Valence	-.10	[-.15, -.05]	< .001	< .001
		ANEW Valence	-.18	[-.23, -.13]	< .001	< .001
		VADER Pos-Neg	-.24	[-.29, -.19]	< .001	< .001
	Fearful face	LIWC Tone	-.02	[-.08, .03]	.352	> .99
		NRC Pos-Neg	-.03	[-.09, .02]	.198	.988
		LS Valence	-.06	[-.11, -.01]	.020	.099
		ANEW Valence	-.10	[-.15, -.05]	< .001	.001
		VADER Pos-Neg	-.19	[-.24, -.14]	< .001	< .001
	Sad face	LIWC Tone	-.03	[-.08, .02]	.236	> .99
		NRC Pos-Neg	-.04	[-.09, .01]	.136	.681
		LS Valence	-.05	[-.10, .00]	.059	.293
		ANEW Valence	-.13	[-.18, -.08]	< .001	< .001
		VADER Pos-Neg	-.12	[-.17, -.07]	< .001	< .001
3b	Happy face	LIWC Tone	.13	[.08, .17]	< .001	< .001
		NRC Pos-Neg	.10	[.06, .15]	< .001	< .001
		LS Valence	.14	[.09, .18]	< .001	< .001
		ANEW Valence	.23	[.19, .28]	< .001	< .001
		VADER Pos-Neg	.26	[.22, .30]	< .001	< .001

	Negative face	LIWC Tone	-.06	[-.10, -.02]	.009	.045
		NRC Pos-Neg	-.10	[-.14, -.05]	< .001	< .001
		LS Valence	-.09	[-.14, -.05]	< .001	< .001
		ANEW Valence	-.27	[-.31, -.23]	< .001	< .001
		VADER Pos-Neg	-.27	[-.31, -.23]	< .001	< .001
	Angry face	LIWC Tone	-.06	[-.10, -.01]	.013	.063
		NRC Pos-Neg	-.08	[-.12, -.03]	< .001	.003
		LS Valence	-.10	[-.14, -.05]	< .001	< .001
		ANEW Valence	-.21	[-.25, -.17]	< .001	< .001
		VADER Pos-Neg	-.26	[-.30, -.21]	< .001	< .001
	Fearful face	LIWC Tone	-.05	[-.10, -.00]	.032	.158
		NRC Pos-Neg	-.06	[-.10, -.01]	.013	.067
		LS Valence	-.07	[-.11, -.02]	.004	.021
		ANEW Valence	-.15	[-.19, -.10]	< .001	< .001
		VADER Pos-Neg	-.24	[-.28, -.20]	< .001	< .001
	Sad face	LIWC Tone	-.02	[-.07, .02]	.308	> .99
		NRC Pos-Neg	-.07	[-.12, -.03]	.002	.012
		LS Valence	-.02	[-.07, .02]	.359	> .99
		ANEW Valence	-.16	[-.20, -.11]	< .001	< .001
		VADER Pos-Neg	-.10	[-.14, -.05]	< .001	< .001

Note. Number of comparisons = 6 for Dataset 2. Number of comparisons = 5 for Dataset 3. CI = confidence interval. Alpha level = .05

Table S4d.

Bonferroni corrected p-values and 95% CIs for correlations between language measures of valence and vocal cues

Dataset	Vocal measure	Language measure	Spearman's rho	95% CI	Original p-value	Bonferroni-corrected p-value
2	Vocal pitch	LIWC Tone	.06	[-.08, .20]	.420	> .99
		NRC Pos-Neg	.02	[-.13, .16]	.828	> .99
		LS Valence	.13	[-.01, .26]	.078	.469
		ANEW Valence	.07	[-.07, .21]	.321	> .99
		VADER Compound	.25	[.11, .38]	< .001	.002
		VADER Pos-Neg	.17	[.03, .31]	.017	.103
	Vocal intensity	LIWC Tone	.00	[-.14, .14]	.998	> .99
		NRC Pos-Neg	-.06	[-.20, .08]	.379	> .99
		LS Valence	.10	[-.05, .23]	.181	> .99
		ANEW Valence	.08	[-.06, .22]	.282	> .99
		VADER Compound	.11	[-.03, .25]	.134	.804
		VADER Pos-Neg	-.01	[-.15, .13]	.864	> .99
3a	Vocal pitch	LIWC Tone	.08	[.03, .13]	.002	.012
		NRC Pos-Neg	-.07	[-.12, -.02]	.006	.029
		LS Valence	.12	[.07, .17]	< .001	< .001
		ANEW Valence	.00	[-.06, .05]	.861	> .99
		VADER Pos-Neg	.04	[-.01, .10]	.094	.470
	Vocal intensity	LIWC Tone	.00	[-.05, .06]	.884	> .99
		NRC Pos-Neg	-.06	[-.11, -.01]	.018	.090
		LS Valence	.05	[.00, .10]	.045	.225
		ANEW Valence	.00	[-.06, .05]	.886	> .99
		VADER Pos-Neg	-.04	[-.10, .01]	.093	.464
3b	Vocal pitch	LIWC Tone	.08	[.03, .12]	< .001	.003
		NRC Pos-Neg	-.03	[-.07, .02]	.249	> .99
		LS Valence	.14	[.09, .18]	< .001	< .001
		ANEW Valence	.06	[.01, .10]	.011	.053
		VADER Pos-Neg	.14	[.09, .18]	< .001	< .001

	Vocal intensity	LIWC Tone	-.02	[-.07, .02]	.285	> .99
		NRC Pos-Neg	-.04	[-.08, .01]	.093	.466
		LS Valence	.05	[.01, .10]	.026	.130
		ANEW Valence	.04	[-.01, .08]	.107	.534
		VADER Pos-Neg	.00	[-.05, .04]	.903	> .99

Note. Number of comparisons = 6 for Dataset 2. Number of comparisons = 5 for Dataset 3. CI = confidence interval. Alpha level = .05

Table S5a.

Spearman correlations between language measures of anger and other measures in Dataset 1 (social rejector narratives)

	1.	2.	3.	4.	5.
1. Pos-Neg Self-Report	—	-.41*	-.11*	-.14*	-.02
2. Frustration Self-Report		—	.29*	.13*	.13*
3. LIWC Anger			—	.27*	.31*
4. NRC Anger				—	.07
5. NRC Anger Intensity					—

*Note. N*s = 496-602. Asterisks indicate $p < .05$.

Table S5b.

Spearman correlations between language measures of fear and other measures in Dataset 1 (social rejector narratives)

	1.	2.	3.	4.	5.
1. Pos-Neg Self-Report	—	-.50*	-.18*	-.13*	.13*
2. Worry Self-Report		—	.24*	.13*	.05
3. LIWC Anxiety			—	.28*	.22*
4. NRC Fear				—	.14*
5. NRC Fear Intensity					—

Note. *N*s = 516-602. Asterisks indicate $p < .05$.

Table S5c.

Spearman correlations between language measures of sadness and other measures in Dataset 1 (social rejector narratives)

	1.	2.	3.	4.	5.
1. Pos-Neg Self-Report	—	-.50*	-.17*	-.13*	-.09*
2. Hurt Self-Report		—	.22*	.19*	.16*
3. LIWC Sadness			—	.32*	.20*
4. NRC Sadness				—	-.14*
5. NRC Sadness Intensity					—

Note. *N*s = 574-602. Asterisks indicate $p < .05$.

Table S5d.

Spearman correlations between language measures of anger and other measures in Dataset 2 (SEND narratives)

	1.	2.	3.	4.	5.	6.	7.	8.
1. Self-Report	—	.80*	-.19*	.07	.08	-.23*	-.27*	-.27*
2. Observer Report		—	-.15*	.13	.10	-.38*	-.34*	-.37*
3. Angry Face			—	-.39*	.03	.10	-.01	-.02
4. Vocal Pitch				—	.43*	-.11	.01	-.18*
5. Vocal Intensity					—	-.04	-.05	.06
6. LIWC Anger						—	.32*	.21*
7. NRC Anger							—	.17*
8. NRC Anger Intensity								—

*Note. N*s = 169-193. Asterisks indicate $p < .05$.

Table S5e.

Spearman correlations between language measures of fear and other measures in Dataset 2 (SEND narratives)

	1.	2.	3.	4.	5.	6.	7.	8.
1. Self-Report	—	.80*	-.09	.07	.08	-.03	-.26*	-.25*
2. Observer Report		—	-.09	.13	.10	-.06	-.30*	-.33*
3. Fearful Face			—	-.16*	-.10	.02	.13	-.03
4. Vocal Pitch				—	. 43*	.12	.05	-.11
5. Vocal Intensity					—	.02	-.09	-.09
6. LIWC Anxiety						—	.21*	.26*
7. NRC Fear							—	.28*
8. NRC Fear Intensity								—

Note. *N*s = 179-193. Asterisks indicate $p < .05$.

Table S5f.

Spearman correlations between language measures of sadness and other measures in Dataset 2 (SEND narratives)

	1.	2.	3.	4.	5.	6.	7.	8.
1. Self-Report	—	.80*	-.14	.07	.08	-.34*	-.37*	-.43*
2. Observer Report		—	-.24*	.13	.10	-.36*	-.46*	-.48*
3. Sad Face			—	-.36*	.00	.00	-.08	.15*
4. Vocal Pitch				—	.43*	-.11	-.03	-.21*
5. Vocal Intensity					—	-.10	-.15*	.02
6. LIWC Sadness						—	.35*	.47*
7. NRC Sadness							—	.18*
8. NRC Sadness Intensity								—

*Note. N*s = 184-193. Asterisks indicate $p < .05$.

Table S5g.

Spearman correlations between language measures of anger and other measures in Dataset 3 (CANDOR conversations)

	1.	2.	3.	4.	5.	6.	7.	8.	9.
1. Pre-Task Self-Report	—	.35*	.40*	.13*	-.02	.01	-.05	-.02	-.01
2. Retrospective Self-Report	.37*	—	.77*	.13*	.03	.06*	.02	.05	.03
3. Post-Task Self-Report	.44*	.84*	—	.14*	.05	.09*	.03	.06*	.05
4. Angry Face	.09*	.08*	.07*	—	-.17*	-.12*	-.04	.01	-.04
5. Vocal Pitch	-.06*	.05*	.07*	-.19*	—	.79*	.08*	-.02	.08*
6. Vocal Intensity	-.02	.10*	.12*	-.12*	.80*	—	.08*	-.01	.05*
7. LIWC Anger	-.03	.03	.03	-.03	.06*	.05*	—	.30*	.30*
8. NRC Anger	-.02	.04	.03	.02	.00	-.01	.32*	—	.11*
9. NRC Anger Intensity	-.02	.03	.02	-.01	.07*	.03	.26*	.12*	—

Note. First-time speakers (Dataset 3a) above the diagonal (Ns = 1406-1455). Returning speakers (Dataset 3b) below the diagonal (Ns = 1837-1856). Asterisks indicate $p < .05$.

Table S5h.

Spearman correlations between language measures of fear and other measures in Dataset 3 (CANDOR conversations)

	1.	2.	3.	4.	5.	6.	7.	8.	9.
1. Pre-Task Self-Report	—	.35*	.40*	.13*	-.02	.01	-.06*	-.02	.02
2. Retrospective Self-Report	.37*	—	.77*	.13*	.03	.06*	.05*	.01	.02
3. Post-Task Self-Report	.44*	.84*	—	.13*	.05	.09*	.04	.03	.04
4. Fearful Face	.08*	.11*	.09*	—	.01	.04	-.01	.02	.03
5. Vocal Pitch	-.06*	.05*	.07*	-.04	—	.79*	.15*	.09*	.03
6. Vocal Intensity	-.02	.10*	.12*	.02	.80*	—	.14*	.06*	.02
7. LIWC Anxiety	-.09*	.03	.01	.04	.19*	.18*	—	.23*	.27*
8. NRC Fear	-.07*	.03	.01	.06*	.07*	.05*	.27*	—	.28*
9. NRC Fear Intensity	-.04	.01	-.01	.03	.00	.03	.30*	.29*	—

Note. First-time speakers (Dataset 3a) above the diagonal (*N*s = 1406-1455). Returning speakers (Dataset 3b) below the diagonal (*N*s = 1837-1856). Asterisks indicate $p < .05$.

Table S5i.

Spearman correlations between language measures of sadness and other measures in Dataset 3 (CANDOR conversations)

	1.	2.	3.	4.	5.	6.	7.	8.	9.
1. Pre-Task Self-Report	—	.35*	.40*	.05	-.02	.01	-.02	-.02	-.05*
2. Retrospective Self-Report	.37*	—	.77*	.02	.03	.06*	.00	.02	.00
3. Post-Task Self-Report	.44*	.84*	—	.03	.05	.09*	.04	.04	.01
4. Sad Face	.04	.03	.01	—	-.08*	-.07*	.03	.07*	.02
5. Vocal Pitch	-.06*	.05*	.07*	-.09*	—	.79*	.12*	.07*	.10*
6. Vocal Intensity	-.02	.10*	.12*	-.09*	.80*	—	.13*	.05	.09*
7. LIWC Sadness	-.03	.06*	.05*	-.02	.09*	.07*	—	.24*	.32*
8. NRC Sadness	-.09*	-.01	-.02	.06*	.05	.05*	.23*	—	.24*
9. NRC Sadness Intensity	-.04	.02	-.01	.02	.06*	.06*	.33*	.23*	—

Note. First-time speakers (Dataset 3a) above the diagonal (*N*s = 1406-1455). Returning speakers (Dataset 3b) below the diagonal (*N*s = 1837-1856). Asterisks indicate $p < .05$.

Table S6a.

Bonferroni corrected p-values and 95% CIs for correlations between language measures of discrete emotions and self-report

Dataset	Self-report measure	Language measure	Spearman's rho	95% CI	Original p-value	Bonferroni-corrected p-value
1	Pos-neg self-report	LIWC Anger	-.11	[-.19, -.03]	.007	.059
		NRC Anger	-.14	[-.21, -.06]	< .001	.006
		NRC Anger Intensity	-.02	[-.11, .06]	.601	> .99
		LIWC Anxiety	-.18	[-.26, -.10]	< .001	< .001
		NRC Fear	-.13	[-.21, -.05]	.001	.012
		NRC Fear Intensity	-.13	[-.22, -.05]	.002	.021
		LIWC Sadness	-.17	[-.24, -.09]	< .001	< .001
		NRC Sadness	-.13	[-.21, -.05]	.001	.009
		NRC Sadness Intensity	-.09	[-.17, -.01]	.034	.303
	Frustration self-report	LIWC Anger	.29	[.17, .32]	< .001	< .001
		NRC Anger	.13	[.02, .18]	.001	.004
		NRC Anger Intensity	.13	[.09, .26]	.004	.011
	Worry self-report	LIWC Anxiety	.24	[.16, .31]	< .001	< .001
		NRC Fear	.13	[.05, .20]	.002	.006
		NRC Fear Intensity	.05	[-.04, .13]	.272	> .99
	Hurt self-report	LIWC Sadness	.22	[.14, .29]	< .001	< .001
		NRC Sadness	.19	[.11, .26]	< .001	< .001
		NRC Sadness Intensity	.16	[.08, .24]	< .001	< .001
2	Self-report	LIWC Anger	-.23	[-.36, -.09]	.001	.012
		NRC Anger	-.27	[-.39, -.13]	< .001	.002
		NRC Anger Intensity	-.27	[-.41, -.13]	< .001	.003
		LIWC Anxiety	-.03	[-.17, .11]	.653	> .99
		NRC Fear	-.26	[-.38, -.12]	< .001	.003
		NRC Fear Intensity	-.25	[-.38, -.10]	.001	.007
		LIWC Sadness	-.34	[-.45, -.20]	< .001	< .001
		NRC Sadness	-.37	[-.48, -.24]	< .001	< .001
		NRC Sadness Intensity	-.43	[-.54, -.31]	< .001	< .001

3a	Pre-task self-report	LIWC Anger	-.05	[-.10, .00]	.056	.507
		NRC Anger	.02	[-.03, .07]	.476	> .99
		NRC Anger Intensity	-.01	[-.06, .04]	.641	> .99
		LIWC Anxiety	-.06	[-.11, -.01]	.019	.169
		NRC Fear	-.02	[-.07, .03]	.392	> .99
		NRC Fear Intensity	.02	[-.04, .07]	.567	> .99
		LIWC Sadness	-.02	[-.07, .03]	.417	> .99
		NRC Sadness	-.02	[-.07, .03]	.479	> .99
		NRC Sadness Intensity	-.05	[-.11, -.00]	.040	.357
	Retrospective self-report	LIWC Anger	.02	[-.03, .07]	.482	> .99
		NRC Anger	.05	[-.01, .10]	.090	.806
		NRC Anger Intensity	.03	[-.02, .08]	.249	> .99
		LIWC Anxiety	.05	[.00, .10]	.048	.429
		NRC Fear	.01	[-.04, .07]	.595	> .99
		NRC Fear Intensity	.02	[-.03, .07]	.431	> .99
		LIWC Sadness	.00	[.05, .08]	.924	> .99
		NRC Sadness	.02	[-.03, .08]	.364	> .99
		NRC Sadness Intensity	.00	[-.05, .05]	.943	> .99
	Post-task self-report	LIWC Anger	.03	[-.02, .09]	.191	> .99
		NRC Anger	.06	[.01, .11]	.024	.212
		NRC Anger Intensity	.05	[-.00, .10]	.070	.626
		LIWC Anxiety	.04	[-.01, .09]	.106	.958
		NRC Fear	.03	[-.02, .08]	.269	> .99
		NRC Fear Intensity	.04	[-.01, .09]	.160	> .99
		LIWC Sadness	.04	[-.01, .09]	.159	> .99
		NRC Sadness	.04	[-.01, .09]	.118	> .99
		NRC Sadness Intensity	.01	[-.04, .06]	.746	> .99
3b	Pre-task self-report	LIWC Anger	-.03	[-.08, .01]	.159	> .99
		NRC Anger	-.02	[-.07, .02]	.335	> .99
		NRC Anger Intensity	-.02	[-.07, .02]	.375	> .99
		LIWC Anxiety	-.09	[-.14, -.05]	< .001	< .001
		NRC Fear	-.07	[-.11, -.02]	.003	.026

	NRC Fear Intensity	-.04	[-.09, .00]	.059	.527
	LIWC Sadness	-.03	[-.08, .01]	.172	> .99
	NRC Sadness	-.09	[-.14, -.05]	< .001	< .001
	NRC Sadness Intensity	-.04	[-.09, .00]	.074	.666
Retrospective self-report	LIWC Anger	.03	[-.02, .07]	.239	> .99
	NRC Anger	.04	[-.01, .08]	.112	> .99
	NRC Anger Intensity	.03	[-.02, .07]	.248	> .99
	LIWC Anxiety	.03	[-.02, .07]	.228	> .99
	NRC Fear	.03	[-.01, .08]	.179	> .99
	NRC Fear Intensity	.01	[-.04, .05]	.795	> .99
	LIWC Sadness	.06	[.02, .11]	.006	.058
	NRC Sadness	-.01	[-.05, .06]	.707	> .99
	NRC Sadness Intensity	.02	[-.03, .06]	.420	> .99
Post-task self-report	LIWC Anger	.03	[-.02, .07]	.201	> .99
	NRC Anger	.03	[-.02, .07]	.263	> .99
	NRC Anger Intensity	.02	[-.03, .06]	.438	> .99
	LIWC Anxiety	.01	[-.04, .05]	.701	> .99
	NRC Fear	.01	[-.04, .05]	.727	> .99
	NRC Fear Intensity	-.01	[-.05, .04]	.767	> .99
	LIWC Sadness	.05	[.01, .10]	.019	.168
	NRC Sadness	-.02	[-.07, .02]	.288	> .99
	NRC Sadness Intensity	-.01	[-.05, .04]	.808	> .99

Note. Number of comparisons = 3 for the frustration, worry, and hurt measures in Dataset 1. Number of comparisons = 9 for the positive – negative measure in Dataset 1 and for Datasets 2 and 3. CI = confidence interval. Alpha level = .05

Table S6b.

Bonferroni corrected p-values and 95% CIs for correlations between language measures of discrete emotions and observer report

Dataset	Observer report measure	Language measure	Spearman's rho	95% CI	Original p-value	Bonferroni-corrected p-value
2	Observer report	LIWC Anger	-.38	[-.49, -.25]	< .001	< .001
		NRC Anger	-.34	[-.46, -.20]	< .001	< .001
		NRC Anger Intensity	-.37	[-.50, -.23]	< .001	< .001
		LIWC Anxiety	-.06	[-.20, .08]	.429	> .99
		NRC Fear	-.30	[-.43, -.17]	< .001	< .001
		NRC Fear Intensity	-.33	[-.46, -.20]	< .001	< .001
		LIWC Sadness	-.36	[-.48, -.23]	< .001	< .001
		NRC Sadness	-.46	[-.57, -.34]	< .001	< .001
		NRC Sadness Intensity	-.48	[-.59, -.36]	< .001	< .001

Note. Number of comparisons = 9. CI = confidence interval. Alpha level = .05

Table S6c.

Bonferroni corrected p-values and 95% CIs for correlations between language measures of discrete emotions and facial cues

Dataset	Facial cue measure	Language measure	Spearman's rho	95% CI	Original p-value	Bonferroni-corrected p-value
2	Angry face	LIWC Anger	.10	[-.04, .24]	.177	.531
		NRC Anger	-.01	[-.15, .13]	.864	> .99
		NRC Anger Intensity	-.02	[-.17, .13]	.773	> .99
	Fearful face	LIWC Anxiety	.02	[-.12, .16]	.756	> .99
		NRC Fear	.13	[-.01, .27]	.072	.215
		NRC Fear Intensity	-.03	[-.17, .12]	.720	> .99
	Sad face	LIWC Sadness	.00	[-.15, .14]	.951	> .99
		NRC Sadness	-.08	[-.22, .06]	.260	.780
		NRC Sadness Intensity	.15	[.01, .29]	.041	.122
3a	Angry face	LIWC Anger	-.04	[-.09, .01]	.113	.3375
		NRC Anger	.01	[-.04, .06]	.683	> .99
		NRC Anger Intensity	-.04	[-.09, .01]	.139	.416
	Fearful face	LIWC Anxiety	-.01	[-.06, .04]	.672	> .99
		NRC Fear	.02	[-.03, .08]	.359	> .99
		NRC Fear Intensity	.03	[-.02, .08]	.235	.706
	Sad face	LIWC Sadness	.03	[-.03, .08]	.243	.729
		NRC Sadness	.07	[.02, .13]	.004	.013
		NRC Sadness Intensity	.02	[-.03, .07]	.449	> .99
3b	Angry face	LIWC Anger	-.03	[-.07, .02]	.218	.653
		NRC Anger	.02	[-.03, .07]	.390	> .99
		NRC Anger Intensity	-.01	[-.06, .03]	.639	> .99
	Fearful face	LIWC Anxiety	.04	[-.00, .09]	.063	.190
		NRC Fear	.06	[.01, .10]	.012	.037
		NRC Fear Intensity	.03	[-.02, .07]	.274	.822
	Sad face	LIWC Sadness	-.02	[-.07, .02]	.344	> .99
		NRC Sadness	.06	[.01, .10]	.013	.039
		NRC Sadness Intensity	.02	[-.02, .07]	.283	.848

Note. Number of comparisons = 3. CI = confidence interval. Alpha level = .05

Table S6d.

Bonferroni corrected p-values and 95% CIs for correlations between language measures of discrete emotions and vocal cues

Dataset	Vocal measure	Language measure	Spearman's rho	95% CI	Original p-value	Bonferroni-corrected p-value
2	Vocal pitch	LIWC Anger	-.11	[-.25, .03]	.124	> .99
		NRC Anger	.01	[-.13, .16]	.839	> .99
		NRC Anger Intensity	-.18	[-.32, -.03]	.018	.161
		LIWC Anxiety	.12	[-.03, .25]	.107	.959
		NRC Fear	.05	[-.09, .19]	.506	> .99
		NRC Fear Intensity	-.11	[-.25, .04]	.148	> .99
		LIWC Sadness	-.11	[-.25, .03]	.128	> .99
		NRC Sadness	-.03	[-.17, .12]	.728	> .99
		NRC Sadness Intensity	-.21	[-.34, -.07]	.005	.041
	Vocal intensity	LIWC Anger	-.04	[-.18, .10]	.539	> .99
		NRC Anger	-.05	[-.19, .09]	.492	> .99
		NRC Anger Intensity	.06	[-.09, .21]	.433	> .99
		LIWC Anxiety	.02	[-.12, .16]	.765	> .99
		NRC Fear	-.09	[-.23, .05]	.229	> .99
		NRC Fear Intensity	-.09	[-.23, .06]	.233	> .99
		LIWC Sadness	-.10	[-.24, .04]	.148	> .99
		NRC Sadness	-.15	[-.28, -.00]	.043	.391
		NRC Sadness Intensity	.02	[-.12, .16]	.782	> .99
3a	Vocal pitch	LIWC Anger	.08	[.03, .13]	.002	.014
		NRC Anger	-.02	[-.07, .03]	.495	> .99
		NRC Anger Intensity	.08	[.03, .14]	.001	.012
		LIWC Anxiety	.15	[.10, .20]	< .001	< .001
		NRC Fear	.09	[.04, .14]	.001	.006
		NRC Fear Intensity	.03	[-.02, .08]	.207	> .99
		LIWC Sadness	.12	[.07, .18]	< .001	< .001
		NRC Sadness	.07	[.02, .13]	.005	.041
		NRC Sadness Intensity	.10	[.04, .15]	< .001	.002

	Vocal intensity	LIWC Anger	.08	[.03, .13]	.002	.018
		NRC Anger	-.01	[-.07, .04]	.579	> .99
		NRC Anger Intensity	.05	[.00, .10]	.046	.412
		LIWC Anxiety	.14	[.09, .19]	< .001	< .001
		NRC Fear	.06	[.01, .11]	.029	.260
		NRC Fear Intensity	.02	[-.03, .07]	.444	> .99
		LIWC Sadness	.13	[.08, .18]	< .001	< .001
		NRC Sadness	.05	[-.00, .10]	.051	.455
		NRC Sadness Intensity	.09	[.04, .15]	< .001	.003
3b	Vocal pitch	LIWC Anger	.06	[.01, .10]	.014	.122
		NRC Anger	.00	[-.05, .04]	.903	> .99
		NRC Anger Intensity	.07	[.02, .11]	.004	.035
		LIWC Anxiety	.19	[.15, .23]	< .001	< .001
		NRC Fear	.07	[.02, .11]	.005	.041
		NRC Fear Intensity	.00	[-.04, .05]	.959	> .99
		LIWC Sadness	.09	[.04, .13]	< .001	.001
		NRC Sadness	.05	[-.00, .09]	.050	.451
		NRC Sadness Intensity	.06	[.01, .10]	.016	.146
	Vocal intensity	LIWC Anger	.05	[.01, .10]	.020	.180
		NRC Anger	-.01	[-.05, .04]	.720	> .99
		NRC Anger Intensity	.03	[-.02, .07]	.210	> .99
		LIWC Anxiety	.18	[.14, .23]	< .001	< .001
		NRC Fear	.05	[.01, .10]	.022	.198
		NRC Fear Intensity	.03	[-.02, .07]	.256	> .99
		LIWC Sadness	.07	[.02, .11]	.004	.036
		NRC Sadness	.05	[.00, .09]	.050	.446
		NRC Sadness Intensity	.06	[.01, .10]	.012	.110

Note. Number of comparisons = 9. CI = confidence interval. Alpha level = .05

Table S7a.

Paired t-test comparisons of patterns of associations across datasets between language measures of valence

Language measure 1	Language measure 2	t	df	Original p-value	Bonferroni-corrected p-value	Effect size (Cohen's d)	95% CI
LIWC Tone	NRC Pos-Neg	3.66	20	.002	.015	.80	[.30, 1.29]
LIWC Tone	LS Valence	-2.78	20	.012	.115	.61	[.13, 1.07]
LIWC Tone	ANEW Valence	1.38	20	.184	> .99	.30	[-.14, .73]
LIWC Tone	VADER Pos-Neg	1.40	20	.177	> .99	.31	[-.14, .74]
NRC Pos-Neg	LS Valence	-4.37	20	< .001	.003	.95	[.43, 1.46]
NRC Pos-Neg	ANEW Valence	-.721	20	.479	> .99	.16	[-.27, .59]
NRC Pos-Neg	VADER Pos-Neg	-.397	20	.695	> .99	.09	[-.34, .51]
LS Valence	ANEW Valence	3.00	20	.007	.071	.65	[.18, 1.12]
LS Valence	VADER Pos-Neg	2.76	20	.012	.119	.60	[.13, 1.06]
ANEW Valence	VADER Pos-Neg	.330	20	.745	> .99	.07	[-.36, .50]

Note. Number of comparisons = 10. Alpha level = .05. N = 21 associations per language measure: Dataset 1 self-report; Dataset 2 self-report, observer report, happy facial cues, negative facial cues, vocal pitch, vocal intensity; Dataset 3a pre-task self-report, retrospective self-report, post-task self-report, happy facial cues, negative facial cues, vocal pitch, vocal intensity; Dataset 3b pre-task self-report, retrospective self-report, post-task self-report, happy facial cues, negative facial cues, vocal pitch, vocal intensity.

Table S7b.

Spearman correlations between patterns of associations across datasets for language measures of valence

	1.	2.	3.	4.	5.
1. LIWC Tone	—	.74*	.91*	.81*	.89*
2. NRC Pos-Neg		—	.64*	.65*	.67*
3. LS Valence			—	.91*	.96*
4. ANEW Valence				—	.97*
5. VADER Pos-Neg					—

Note. $N = 21$. Asterisks indicate $p < .05$. N = 21 associations per language measure: Dataset 1 self-report; Dataset 2 self-report, observer report, happy facial cues, negative facial cues, vocal pitch, vocal intensity; Dataset 3a pre-task self-report, retrospective self-report, post-task self-report, happy facial cues, negative facial cues, vocal pitch, vocal intensity; Dataset 3b pre-task self-report, retrospective self-report, post-task self-report, happy facial cues, negative facial cues, vocal pitch, vocal intensity.

Table S8.

Paired t-test comparisons of patterns of associations across datasets between language measures of discrete emotions

Language measure 1	Language measure 2	t	df	Original p-value	Bonferroni-corrected p-value	Effect size (Cohen's d)	95% CI
LIWC Anger	NRC Anger	.808	18	.429	> .99	.19	[-.27, .64]
LIWC Anger	NRC Anger Intensity	.569	18	.577	> .99	.13	[-.32, .58]
NRC Anger	NRC Anger Intensity	-.322	18	.751	> .99	.07	[-.38, .52]
LIWC Anxiety	NRC Fear	2.32	18	.032	.097	.53	[.04, 1.01]
LIWC Anxiety	NRC Fear Intensity	3.30	18	.004	.012	.76	[.24, 1.26]
NRC Fear	NRC Fear Intensity	2.54	18	.021	.062	.58	[.09, 1.06]
LIWC Sadness	NRC Sadness	1.77	18	.094	.283	.40	[-.07, .87]
LIWC Sadness	NRC Sadness Intensity	.842	18	.411	> .99	.19	[-.26, .64]
NRC Sadness	NRC Sadness Intensity	-.422	18	.678	> .99	.10	[-.36, .55]

Note. Number of comparisons = 3. Alpha level = .05. N = 19 associations per language measure: Dataset 1 valence self-report, discrete self-report (frustration, worry, or hurt); Dataset 2 self-report, observer report, discrete facial cues (angry, fearful, or sad), vocal pitch, vocal intensity; Dataset 3a pre-task self-report, retrospective self-report, post-task self-report, discrete facial cues (angry, fearful, or sad), vocal pitch, vocal intensity; Dataset 3b pre-task self-report, retrospective self-report, post-task self-report, discrete facial cues (angry, fearful, or sad), vocal pitch, vocal intensity.

Supplementary Note 1.

In addition to language measures of valence and discrete emotions which focus on the extent to which language can be presumed to reflect positive or negative emotions, language dictionaries may also measure the frequency of emotion-related words (i.e., the number of emotion words used) or the intensity of emotion-related words (i.e., the strength of emotion words used, such as *annoyed* versus *furious*). Previous research has not tested associations between language measures of emotion frequency or emotional intensity and other measures often used to assess emotion.

Table S9.

Descriptive statistics for language measures of emotion frequency and emotional intensity in Datasets 1 (rejector narratives), 2 (SEND narratives), and 3 (CANDOR conversations)

	**Dataset 1**			**Dataset 2**			**Dataset 3**		
	M	SD	Range	M	SD	Range	M	SD	Range
**Emotion Frequency**									
LIWC Affect	4.37	1.74	0.00–11.97	4.21	1.53	1.16–10.30	3.78	0.89	1.42–8.25
LIWC Emotion	1.50	1.08	0.00–7.04	1.91	1.06	0.00–6.02	1.34	0.50	0.12–5.73
NRC Pos + Neg	5.95	2.06	0.54–14.05	5.46	1.74	1.39–12.03	4.79	0.81	2.23–8.51
Lexical Suite Pos + Neg	0.02	0.01	0.00–0.08	0.03	0.01	0.00–0.09	0.03	0.01	0.01–0.07

**Emotional Intensity**									
NRC Mean Intensity	0.45	0.06	0.23–0.68	0.45	0.07	0.28–0.62	0.44	0.03	0.29–0.54
LS Extremity	2.65	0.50	0.45–3.95	2.77	0.42	1.31–3.95	2.68	0.19	1.47–3.26
LS Emotionality	4.87	0.76	1.81–7.11	5.16	0.75	2.83–8.26	4.64	0.35	3.07–5.81
ANEW Arousal	5.35	0.23	4.55–6.22	5.49	0.21	4.83–6.08	5.54	0.17	5.03–6.09
VADER Mean Intensity	0.09	0.03	0.00–0.17	0.11	0.03	0.04–0.21	0.16	0.03	0.07–0.29

Note. Each of the emotion frequency language measures reflects the total proportion of positive and negative emotion-related words. NRC Mean Intensity reflects the average intensity score (0 to 1) across positive and negative discrete emotions. VADER Mean Intensity reflects the average of the positive and negative intensity scores (0-1).

Table S10a.

Spearman correlations between language measures of emotion frequency and other measures in Dataset 1 (social rejector narratives)

	1.	2.	3.	4.	5.
1. Self-Report	—	-.13*	-.18*	-.19*	-.10*
2. LIWC Affect		—	.60*	.34*	.52*
3. LIWC Emotion			—	.31*	.49*
4. NRC Pos + Neg				—	.23*
5. Lexical Suite Pos + Neg					—

*Note. N*s = 601-602. Asterisks indicate $p < .05$.

Table S10b.

Spearman correlations between language measures of emotion frequency and other measures in Dataset 2 (SEND narratives)

	1.	2.	3.	4.	5.	6.	7.	8.	9.	10.	11.	12.
1. Self-Report	—	.80*	.17*	-.19*	-.09	-.14	.07	.08	.14	.05	.12	.08
2. Observer Report		—	.25*	-.15*	-.09	-.24*	.13	.10	.15*	.01	.07	.13
3. Happy Face			—	-.23*	.00	-.50*	.25*	.00	.13	.12	.07	.13
4. Angry Face				—	.15*	.38*	-.39*	.03	-.10	-.20*	.02	.07
5. Fearful Face					—	.27*	-.16*	-.10	-.07	-.05	.01	.03
6. Sad Face						—	-.36*	.00	-.12	-.13	-.13	-.10
7. Vocal Pitch							—	.43*	-.01	.04	-.05	-.14
8. Vocal Intensity								—	-.20*	-.23*	-.20*	-.28*
9. LIWC Affect									—	.69*	.42*	.52*
10. LIWC Emotion										—	.45*	.51*
11. NRC Pos + Neg											—	.37*
12. Lexical Suite Pos + Neg												—

Note. N = 193. Asterisks indicate $p < .05$.

Table S10c.

Spearman correlations between language measures of emotion frequency and other measures in Dataset 3 (CANDOR conversations)

	1.	2.	3.	4.	5.	6.	7.	8.	9.	10.	11.	12.	13.
1. Pre-Task Self-Report	—	.35*	.40*	-.08*	.13*	.13*	.05	-.02	.01	-.01	-.02	.00	-.03
2. Retrospective Report	.37*	—	.77*	-.05	.13*	.13*	.02	.03	.06*	.02	.03	.00	.03
3. Post-Task Self-Report	.44*	.84*	—	-.06*	.14*	.13*	.03	.05	.09*	.00	.04	.00	.01
4. Happy Face	-.06*	.02	.03	—	-.50*	-.44*	-.48*	.20*	.14*	.12*	.16*	.03	.12*
5. Angry Face	.09*	.08*	.07*	-.51*	—	.22*	.02	-.17*	-.12*	-.07*	-.09*	-.02	-.06*
6. Fearful Face	.08*	.11*	.09*	-.47*	.27*	—	.27*	.01	.04	-.03	-.02	.00	-.01
7. Sad Face	.04	.03	.01	-.46*	.07*	.27*	—	-.08*	-.07*	.02	.00	.02	.02
8. Vocal Pitch	-.06*	.05*	.07*	.24*	-.19*	-.04	-.09*	—	.79*	.18*	.23*	.00	.13*
9. Vocal Intensity	-.02	.10*	.12*	.15*	-.12*	.02	-.09*	.80*	—	.06*	.13*	-.04	.04
10. LIWC Affect	-.07*	-.02	-.03	.11*	-.07*	-.04	.01	.17*	.04	—	.77*	.48*	.70*
11. LIWC Emotion	-.04	.01	.00	.15*	-.10*	-.05*	-.01	.27*	.14*	.76*	—	.37*	.59*
12. NRC Pos + Neg	-.11*	-.04	-.05*	.04	-.05*	.05*	-.01	.00	-.02	.45*	.35*	—	.35*
13. Lexical Suite Pos + Neg	-.06*	.00	.00	.11*	-.05*	-.03	-.01	.11*	.01	.70*	.61*	.31*	—

Note. First-time speakers (Dataset 3a) above the diagonal (*N*s = 1447-1455). Returning speakers (Dataset 3b) below the diagonal (*N*s = 1851-1856). Asterisks indicate $p < .05$.

Table S11a.

Spearman correlations between language measures of emotional intensity and other measures in Dataset 1 (social rejector narratives)

	1.	2.	3.	4.	5.	6.
1. Self-Report	—	-.04	.03	-.09*	-.01	-.06
2. NRC Mean Intensity		—	.12*	.10*	.17*	.24*
3. LS Extremity			—	.19*	.10*	.06
4. LS Emotionality				—	.18*	.07
5. ANEW Arousal					—	.37*
6. VADER Mean Intensity						—

*Note. N*s = 588-602. Asterisks indicate $p < .05$.

Table S11b.

Spearman correlations between language measures of emotional intensity and other measures in Dataset 2 (SEND narratives)

	1.	2.	3.	4.	5.	6.	7.	8.	9.	10.	11.	12.	13.
1. Self-Report	—	.80*	.17*	-.19*	-.09	-.14	.07	.08	-.29*	.18*	.04	.17*	.04
2. Observer Report		—	.25*	-.15*	-.09	-.24*	.13	.10	-.33*	.22*	-.03	.13	.05
3. Happy Face			—	-.23*	.00	-.50*	.25*	.00	-.12	.07	-.04	.23*	.31*
4. Angry Face				—	.15*	.38*	-.39*	.03	.05	-.04	-.09	-.10	-.15*
5. Fearful Face					—	.27*	-.16*	-.10	-.07	-.08	.07	.10	.00
6. Sad Face						—	-.36*	.00	.07	-.02	.07	-.03	-.14
7. Vocal Pitch							—	.43*	-.16*	.06	-.01	.08	.11
8. Vocal Intensity								—	.03	-.10	-.24*	.05	-.11
9. NRC Mean Intensity									—	-.07	.07	.13	.03
10. LS Extremity										—	.34*	.07	.10
11. LS Emotionality											—	.08	.14
12. ANEW Arousal												—	.55*
13. VADER Mean Intensity													—

Note. $N = 193$. Asterisks indicate $p < .05$.

Table S11c.

Spearman correlations between language measures of emotional intensity and other measures in Dataset 3 (CANDOR conversations)

	1.	2.	3.	4.	5.	6.	7.	8.	9.	10.	11.	12.	13.	14.
1. Pre-Task Self-Report	—	.35*	.40*	-.08*	.13*	.13*	.05	-.02	.01	.00	.09*	-.02	-.15*	-.14*
2. Retrospective Self-Report	.37*	—	.77*	-.05	.13*	.13*	.02	.03	.06*	.05	.10*	.07*	-.04	-.14*
3. Post-Task Self-Report	.44*	.84*	—	-.06*	.14*	.13*	.03	.05	.09*	.07*	.10*	.09*	-.05*	-.13*
4. Happy Face	-.06*	.02	.03	—	-.50*	-.44*	-.48*	.20*	.14*	.02	-.02	.10*	.17*	.25*
5. Angry Face	.09*	.08*	.07*	-.51*	—	.22*	.02	-.17*	-.12*	.01	.07*	-.04	-.20*	-.27*
6. Fearful Face	.08*	.11*	.09*	-.47*	.27*	—	.27*	.01	.04	.06*	.08*	.01	-.09*	-.20*
7. Sad Face	.04	.03	.01	-.46*	.07*	.27*	—	-.08*	-.07*	.04	.06*	-.04	-.11*	-.11*
8. Vocal Pitch	-.06*	.05*	.07*	.24*	-.19*	-.04	-.09*	—	.79*	.10*	-.03	.08*	.05	.12*
9. Vocal Intensity	-.02	.10*	.12*	.15*	-.12*	.02	-.09*	.80*	—	.08*	-.02	.08*	.06*	-.01
10. NRC Mean Intensity	-.04	.03	.02	.00	.01	.03	.01	.08*	.07*	—	.19*	.24*	.07*	.00
11. LS Extremity	.12*	.15*	.15*	-.08*	.11*	.06*	.05*	-.06*	-.04	.16*	—	.31*	-.09*	-.06*
12. LS Emotionality	.01	.10*	.10*	.11*	-.05*	-.01	-.08*	.16*	.13*	.26*	.35*	—	.22*	.16*
13. ANEW Arousal	-.10*	-.02	-.01	.18*	-.21*	-.13*	-.14*	.09*	.10*	.10*	-.11*	.23*	—	.46*

14. VADER Mean Intensity	-.12*	-.10*	-.08*	.27*	-.28*	-.24*	-.09*	.19*	.03	.07*	-.06*	.15*	.50*	—

Note. First-time speakers (Dataset 3a) above the diagonal (*N*s = 1411-1455). Returning speakers (Dataset 3b) below the diagonal (*N*s = 1833-1856). Asterisks indicate $p < .05$.
