# Supplementary material for: Language measures correlate with other measures used to study emotion
Source: Commun Psychol. 2025 Feb 22;3:29. doi: 10.1038/s44271-025-00212-x (PMC11847001; doi:10.1038/s44271-025-00212-x)
Supplement: Supplementary file 3 — Reporting Summary [file 44271_2025_212_MOESM3_ESM.pdf]

nature portfolio

Corresponding author(s): Shaina Munin

Last updated by author(s): Jan 22, 2025

Reporting Summary

Nature Portfolio wishes to improve the reproducibility of the work that we publish. This form provides structure for consistency and transparency in reporting. For further information on Nature Portfolio policies, see our Editorial Policies and the Editorial Policy Checklist.

Statistics

For all statistical analyses, confirm that the following items are present in the figure legend, table legend, main text, or Methods section.

n/a	Confirmed	
☐	☒	The exact sample size (*n*) for each experimental group/condition, given as a discrete number and unit of measurement
☐	☒	A statement on whether measurements were taken from distinct samples or whether the same sample was measured repeatedly
☐	☒	The statistical test(s) used AND whether they are one- or two-sided<br>*Only common tests should be described solely by name; describe more complex techniques in the Methods section.*
☒	☐	A description of all covariates tested
☐	☒	A description of any assumptions or corrections, such as tests of normality and adjustment for multiple comparisons
☐	☒	A full description of the statistical parameters including central tendency (e.g. means) or other basic estimates (e.g. regression coefficient) AND variation (e.g. standard deviation) or associated estimates of uncertainty (e.g. confidence intervals)
☐	☒	For null hypothesis testing, the test statistic (e.g. F, t, r) with confidence intervals, effect sizes, degrees of freedom and P value noted<br>*Give P values as exact values whenever suitable.*
☒	☐	For Bayesian analysis, information on the choice of priors and Markov chain Monte Carlo settings
☒	☐	For hierarchical and complex designs, identification of the appropriate level for tests and full reporting of outcomes
☐	☒	Estimates of effect sizes (e.g. Cohen's *d*, Pearson's *r*), indicating how they were calculated

Our web collection on statistics for biologists contains articles on many of the points above.

Software and code

Policy information about availability of computer code

Data collection	Dataset 1 was collected using Qualtrics. Datasets 2 and 3 were collected by other groups.
Data analysis	Analyses were conducted in R (v. 4.3.2) using the Hmisc (Harrell Jr, 2023) and DescTools (Signorell, 2023) packages. Analysis code can be publicly accessed at https://osf.io/zw3e5/.

For manuscripts utilizing custom algorithms or software that are central to the research but not yet described in published literature, software must be made available to editors and reviewers. We strongly encourage code deposition in a community repository (e.g. GitHub). See the Nature Portfolio guidelines for submitting code & software for further information.

Data

Policy information about availability of data

All manuscripts must include a data availability statement. This statement should provide the following information, where applicable:

- Accession codes, unique identifiers, or web links for publicly available datasets
- A description of any restrictions on data availability
- For clinical datasets or third party data, please ensure that the statement adheres to our policy

The three datasets used in the present work are publicly available (Dataset 1: https://osf.io/v2wpd; Dataset 2: https://github.com/StanfordSocialNeuroscienceLab/SEND; Dataset 3: https://betterup-data-requests.herokuapp.com/).

Human research participants

Policy information about studies involving human research participants and Sex and Gender in Research.

Reporting on sex and gender	Gender was self-reported by participants in all 3 datasets. Data on participant gender is included in the source data and summarized in Table 1 in the manuscript
Population characteristics	See Table 1 in the manuscript for details on participant age, gender, and race in all 3 datasets.
Recruitment	Participants in Dataset 1 were recruited through the undergraduate subject pool and through community recruitment. There may be self-selection biases given that participants were either enrolled in an undergraduate psychology course and/or were interested in participating in a psychology study. For details on recruitment for Datasets 2 and 3, see Ong et al., 2021 and Reece et al., 2023.
Ethics oversight	Study protocols were approved by IRBs at the respective institution for each dataset. Data collection for Dataset 1 was approved by the IRB at The University of Texas at Austin.

Note that full information on the approval of the study protocol must also be provided in the manuscript.

Field-specific reporting

Please select the one below that is the best fit for your research. If you are not sure, read the appropriate sections before making your selection.

☐ Life sciences ☒ Behavioural & social sciences ☐ Ecological, evolutionary & environmental sciences

For a reference copy of the document with all sections, see nature.com/documents/nr-reporting-summary-flat.pdf

Behavioural & social sciences study design

All studies must disclose on these points even when the disclosure is negative.

Study description	Quantitative data, including quantitative analysis of language data.
Research sample	Dataset 1 includes undergraduates and community participants based in the southern US. Dataset 2 (SEND) is an archival dataset of participants based in the West Coast of the US (see Ong et al., 2021). Dataset 3 (CANDOR Corpus) is an archival dataset of participants based in the US (see Reece et al., 2023).
Sampling strategy	Dataset 1 used convenience sampling. The target sample size for Dataset 1 was based on a power analysis for a separate research question using the dataset.<br>Dataset 2: See Ong et al., 2021<br>Dataset 3: See Reece et al., 2023
Data collection	Dataset 1: Data were collected on a computer in an experimental suite. No one was present aside from the participant and experimenter. The experimenter was blind to the study hypotheses.<br>Dataset 2: See Ong et al., 2021<br>Dataset 3: See Reece et al., 2023
Timing	Dataset 1: Data were collected between October 2022 and October 2023<br>Dataset 2: See Ong et al., 2021<br>Dataset 3: See Reece et al., 2023
Data exclusions	No data were excluded from Datasets 1 or 2. One transcript from Dataset 3 (3a) was excluded due to low word count. Missing data were removed pairwise from correlation analyses.
Non-participation	Dataset 1: No participants dropped out or declined participation.<br>Dataset 2: See Ong et al., 2021<br>Dataset 3: See Reece et al., 2023
Randomization	Participants were not allocated into experimental groups.

Reporting for specific materials, systems and methods

We require information from authors about some types of materials, experimental systems and methods used in many studies. Here, indicate whether each material, system or method listed is relevant to your study. If you are not sure if a list item applies to your research, read the appropriate section before selecting a response.

Materials & experimental systems

n/a	Involved in the study
☒	☐ Antibodies
☒	☐ Eukaryotic cell lines
☒	☐ Palaeontology and archaeology
☒	☐ Animals and other organisms
☒	☐ Clinical data
☒	☐ Dual use research of concern

Methods

n/a	Involved in the study
☒	☐ ChIP-seq
☒	☐ Flow cytometry
☒	☐ MRI-based neuroimaging
